# Nanocarriers as Active Ingredients Enhancers in the Cosmetic Industry—The European and North America Regulation Challenges

**DOI:** 10.3390/molecules27051669

**Published:** 2022-03-03

**Authors:** Cristiana Oliveira, Cristina Coelho, José A. Teixeira, Pedro Ferreira-Santos, Claudia M. Botelho

**Affiliations:** 1 CEB—Center of Biological Engineering, University of Minho, Campus de Gualtar, 4710-057 Braga, Portugal; pg42888@alunos.uminho.pt (C.O.); jateixeira@deb.uminho.pt (J.A.T.); 2 LABBELS—Associate Laboratory, Braga/Guimarães, 4710-057 Guimarães, Portugal; 3 Mesosystem S.A., Rua de Júlio Dinis 228, 4050-371 Porto, Portugal; quality2@mesosystem.com

**Keywords:** delivery systems, nanotechnology, cosmetics, legislation, Europe, USA

## Abstract

“Flawless skin is the most universally desired human feature” is an iconic statement by Desmond Morris. Skin indicates one´s health and is so important that it affects a person’s emotional and psychological behavior, these facts having propelled the development of the cosmetics industry. It is estimated that in 2023, this industry will achieve more than 800 billion dollars. This boost is due to the development of new cosmetic formulations based on nanotechnology. Nanocarriers have been able to solve problems related to active ingredients regarding their solubility, poor stability, and release. Even though nanocarriers have evident benefits, they also present some problems related to the high cost, low shelf life, and toxicity. Regulation and legislation are two controversial topics regarding the use of nanotechnology in the field of cosmetics. In this area, the U.S. FDA has taken the lead and recommended several biosafety studies and post-market safety evaluations. The lack of a global definition that identifies nanomaterials as a cosmetic ingredient is a hindrance to the development of global legislation. In the EU, the legislation regarding the biosafety of nanomaterials in cosmetics is stricter. “The cost is not the only important issue, safety and the application of alternative testing methods for toxicity are of crucial importance as well”.

## 1. Introduction

The need to look our best is of utmost importance in society. A well-known pronouncement is that the “eyes are the windows to our soul”, but skin appearance can also tell a lot about a person’s health and state of mind. In 1967, an iconic statement by the zoologist Desmond Morris that “flawless skin is the most universally desired human feature” [1], clearly identifies the importance of healthy skin. Skin appearance indicates a person’s general health status, vitality, and nutritional state [2,3,4,5,6,7,8,9]. Indeed, skin health is related to overall well-being, in other words, the skin is the body’s “visual certificate of health” [9,10]. The importance of skin appearance in dermatological disorders is evident since their clear visibility can significantly influence the patients’ daily activities, mental well-being, self-esteem, and social relationships [11,12,13]. Moreover, skin is a person’s primary interface with their surroundings, so its quality may affect the judgments of others regarding their emotional and psychological health, youthfulness, and personality traits [9,13,14,15]. Therefore, advances in the cosmetics field are extremely important, since the development of new technologies, products, and aesthetic procedures promotes the quality of the skin and, as a result, the general well-being of a person. It has been shown that non-invasive facial rejuvenation allows for sustained improvements in self-ratings of attractiveness and self-esteem and decreases self-perceived age [9,16,17,18,19,20].

It is important to mention that skin, being the major organ of the integumentary system and composed of three main layers (epidermis, dermis, and subcutaneous tissue), plays several roles in human health. Skin is involved in the physical, chemical, and biological protection of internal organs from environmental threats such as the presence of particles, chemicals, dehydration, and infections. Skin also has an important role in the thermo- and hydro-regulation mechanisms [21,22].

In the last years, there has been significant development of the cosmetics industry, which has huge growth potential. In 2017, the global market for the cosmetic industry was evaluated at USD 532.43 billion and is expected to reach 805.61 billion by 2023, with a compound annual growth rate higher than 7% (7.14% 2018 to 2023) [23].

The exponential rise in the cosmetics industry has been driven by the significant demand for personalized and innovative products designed based on ever more detailed scientific knowledge [24,25].

As a definition, a cosmetic product is a substance or mixture of substances that can be used on the external areas of the human body such as the epidermis, hair, lips, nails, external genital organs, teeth, and mucous membranes of the mouth. The aim is to clean, perfume, or protect the site of application, changing its appearance, preserving it, or correcting odors emanating from it [26].

The cosmetic efficacy depends not only on the active ingredients present but also on the technology used to prepare them [25]. Generally, products such as creams, lotions, and gels have active ingredients and substances that form the base, vehicle, and product presentation; each component has a purpose in the formulation. The vehicle purpose is to efficiently transport the active ingredient to the target site and to ensure that it remains at the target location enough time to reach the desired effect [25,27]. The vehicle should ensure the chemical, physical, and microbiological stability of the whole formulation. It is important to mention that the vehicle does not have to be inert, it can also have biological properties with the ability to contribute to the overall effect [25]. For instance, lecithin is widely used as an emulsifier in cosmetic formulations, which has a moisturizing effect, contributing to skin hydration [28].

The cosmetics industry is adopting newer technologies to develop their products, particularly technology at the nano level.

Nanotechnology is a powerful and innovative technology that has revolutionized science in the 21st century [29,30]. A growth rate of 17% (on average) of nanotechnology-based products is expected in the global cosmetics market each year [23]. In general, this technology allows for the manipulation of matter at the nanoscale, which is in the range of 1–100 nm. Nanotechnology allows for the enhancement of several properties such as durability, water resistance, strength, and conductive resistance. This technology enables the production of engineered nanomaterials for several consumer products in different areas such as cosmetics, coatings, food, textiles, medicine, etc. [30]. Concerning the medical field, nanotechnology has played a fundamental role in improved drug delivery systems, leading to the development of new therapies [31,32,33]. In the cosmetics industry, many nano-based products have been developed comprising nanomaterials of different compositions, sizes, and shapes. Indeed, due to the large variety of nanomaterials, it is possible to group them into two broad classes: organic and inorganic nanoparticles. The organic nanoparticles can include lipid-, surfactant-, and polymer-based nanostructures. Among the lipid- and surfactant-derived nanoparticles, there are vesicular and non-vesicular systems. On the other hand, inorganic nanoparticles are composed of metals or metal oxides [25].

These nanomaterials have mostly been chosen due to their ability to overcome the typical limitations of cosmetics such as penetration, stability, and active ingredient-controlled release. This new approach conferred a new potential to the products as the nanomaterials can also be active agents [25,34]. Nanostructures, as the name indicates, have small dimensions with a large surface-to-volume ratio, allowing an increased encapsulation efficiency, the production of formulations with a lighter texture, and better dispersibility and transparency [25,35].

The cosmetics industry aims to innovate, so a significant investment has been made in the field of cosmeceuticals. Cosmeceuticals include personal care products that contain biologically active ingredients with medicinal or druglike benefits [36,37]. It is important to mention that a cosmeceutical does not require Food and Drug Administration (FDA) approval, making it easier to take the developed products from the “bench to the clinics”. This area is the borderline between personal care product cosmetics and pharmaceuticals. Cosmeceuticals satisfy the requirements of beauty and health, carrying out their functions as whitening, anti-wrinkling, antiaging, tanning, protection, deodorants, and hair and nail care [36]. Nanotechnology is very important in this field as it has widely contributed to overcoming the limitations associated with conventional products, leading to a more efficient active ingredient release, therefore increasing the final value of the product meeting the consumers’ needs [37].

This review paper discusses the impact of nanocarriers in the cosmetics industry, namely, their recent applications, efficacy, and legislation challenges in Europe and the USA.

## 2. Nanotechnology in Cosmetology

**Figure 1:**
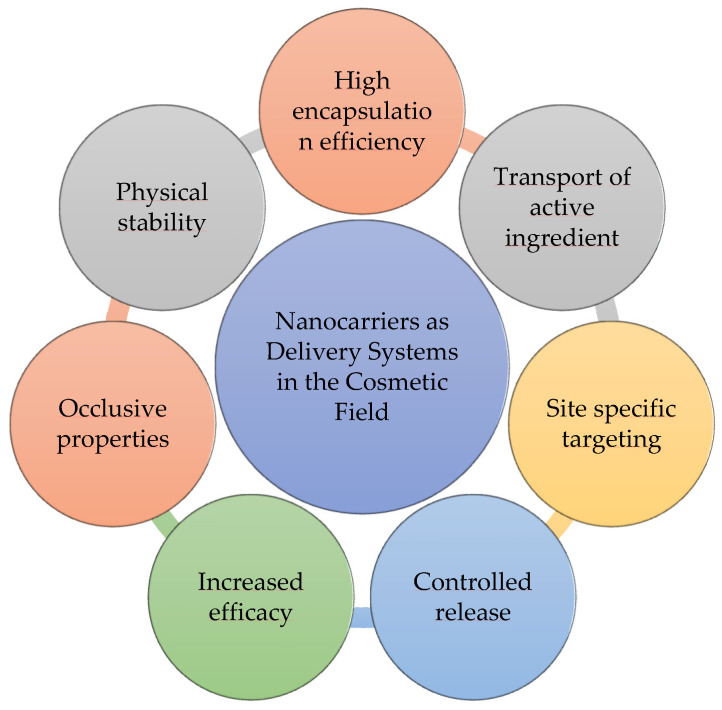
Advantages of using nanocarriers as delivery systems in the cosmetics industry.

Nanotechnology has been implemented in the cosmetics industry for more than 30 years [38,39,40]. The low solubility penetration, poor stability, or uncontrolled release of cosmetic active ingredients can be improved by the use of nanocarriers [41,42,43,44]. As Davies et al. reported, the co-nanoencapsulation of resveratrol and lipoic acid increased their chemical stability, photostability, antioxidant activity, and skin permeation [45]. The nanoencapsulation also resulted in a more controlled release through the lipid-core nanocapsules [45]. The use of nanotechnology is crucial for the size reduction in the formulation ingredients, improving deep skin penetration, sustained skin absorption, ultraviolet (UV) protection, higher stability, and the final quality of the product [46]. In fact, nanotechnology-based formulations have been frequently used, not only in different beauty products and skincare products, but also in sunscreens, hair care products, deodorants, perfumes, and dental products, since they enhance the performance of the active ingredients. In summary, nanocarriers have been used as delivery systems to improve the efficiency of cosmetic products (Figure 1).

Several studies have demonstrated the benefits of nanotechnology in the cosmetics field. Chaki et al. [47] reported that the use of yttrium oxide deposited onto titanium dioxide nanoparticles, forming yttria based nanocomposites, had improved optical and biocompatibility properties with reduced photocatalytic activity, making them suitable for use in sunscreen products. Cyclosporine A is commonly used as a cosmetic ingredient, although its high molecular weight and poor water solubility limits its topical administration. A recent study by Silva et al. [48] revealed that the incorporation of cyclosporine A into solid lipid nanoparticles, Softisan^®^ 649, subjected to a freeze-drying process resulted in an oleogel with pseudoplastic behaviors, leading to an in vitro controlled permeation profile. Moreover, this delivery system allows for the direct application of these ingredients onto the skin, discarding the incorporation of the nanoparticles into a gel, cream, or ointment, which is an advantage over conventional solid lipid nanoparticles.

Phenolic compounds, due to their numerous properties such as antioxidant, antimicrobial, and anti-inflammatory can be used to overcome several problems such as skin aging, pigmentation disorders, solar exposure effects, and cancer [49,50,51]. However, these compounds are not stable upon extraction, being susceptible to degradation, resulting in low bioavailability [52]. They are also rapidly metabolized and poorly soluble in water. Therefore, to take advantage of all of their biological properties, it is necessary to overcome the above-described problems, which is through encapsulation into nanocarriers such as liposomes [53]. Furthermore, Kalouta et al. [54] showed that the undesirable sensory characteristics of natural extracts can be overcome by nanoencapsulation, allowing them to be incorporated in cosmeceutical facial creams. Cycloastragenol is a saponin plant that acts as a telomerase activator and has been used as an oral anti-aging supplement and as an active ingredient in topical cosmetic formulations. However, its direct topical application is not yet possible as its penetration across the skin barrier has not been proven. Therefore, to overcome the low or no existence skin permeability, Wang et al. [55] prepared phospholipid vesicles such as liposomes, transethosomes, and ethosomes using soy and sunflower phospholipids with different penetration enhancers (ethanol and surfactants) to deliver cycloastragenol across the skin barrier. This study showed that the encapsulation of cycloastragenol molecules into phospholipid vesicles enhanced its transport through the skin.

In brief, the ultimate goal of the cosmetics industry is to develop the most efficient formulation. Therefore, nanotechnology is a strong ally for them to achieve this goal as nanotechnology-based materials improve the delivery rate of the active ingredients to the target site with long-term stability [56]. In this way, world-famous cosmetics brands are increasingly using nanocarriers in their products [57]. L’Oréal S.A, a well-known cosmetic brand, ranks sixth place in the United States regarding nanotechnology-related patents, just by essentially using four nano-ingredients, TiO_2_, ZnO, silica, and carbon black, in their products. Another example is the Shiseido Company, which uses TiO_2_ and ZnO nanoparticles in wet-based formulas such as emulsions [56].

## 3. Nanocarriers

Nanocarriers, whose main function is to transport and deliver bioactive agents to a target tissue [58,59,60,61], can be composed of several materials with different structures. The primary characteristic of a nanocarrier is its size. The size of the particle is of utmost importance as it influences the biological properties of the carrier [62]. Indeed, the size influences the penetration ability and cellular uptake of nanocarriers as well as the encapsulation, blood circulation time, pharmacokinetics, and pharmacodynamics [63]. Furthermore, the physical and chemical properties of bioactive molecules as well as their biological characteristics can be altered upon loading them into nanocarriers. This effect is particularly due to the preparation methods such as dissolution, dispersion, encapsulation, adsorption, and coupling. In fact, properties such as saturation, solubility, dissolution rate, crystal characteristics, hydrophilic and hydrophobic, stability, specific molecular affinity, cell affinity, and biodegradability can be modified by nanoencapsulation. In turn, these modifications can positively affect the absorption, distribution, metabolism, and excretion of active components. Moreover, the therapeutic effect and bioavailability of cosmetic efficacy components can be intensified, and its adverse reactions can be attenuated by encapsulation [62]. Given the positive impact of nanocarriers on the improvement of cosmetic products, various novel carrier systems and nanomaterials have been developed (Table 1).

Several nanostructures can be used in the cosmetic field, some of are briefly described in Table 1 as well as some characteristics of their composition and sizes.

In the next section, we discuss the main characteristics and applications of the carriers listed in the previous table (Table 1).

### 3.1. Nanoemulsions

**Figure 2:**
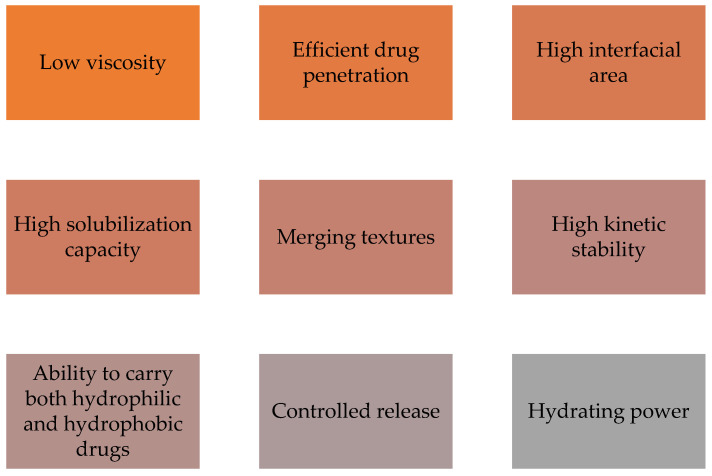
Properties of the nanoemulsions.

A nanoemulsion is transparent or translucent with good properties, as described in Figure 2 such as low viscosity, efficient drug penetration, elevated interfacial area, high solubilization capacity, merging textures, and high kinetic stability as well as the ability to carry both hydrophilic and hydrophobic drugs, controlled release, and hydrating power. These characteristics make this type of nanocarrier a suitable candidate for delivering cosmetic ingredients to the skin [64,65]. A study reported by Kong et al. [75] revealed that lipophilic hyaluronic acid can be carried by nanoemulsions and effectively used as a transdermal delivery system for cosmetic applications. Furthermore, it has been shown by Kabri et al. [76] that a nanoemulsion with salmon oil, miglyol, and rapeseed oil as a matrix is a cosmetic transdermal formulation with extremely good characteristics regarding its turbidity, stability, and size. Arianto et al. [77] prepared sunflower oil nanoemulsions by the spontaneous emulsification method. Arianto and colleagues showed that the sunflower oil nanoemulsion with a ratio of Tween 80 and sorbitol of 38:22 had a higher sun protection factor compared to an emulsion. Therefore, this nanoemulsion formulation is considered to be more efficient for sunscreen cosmetic use than the emulsion [77]. Additionally, Kazemi et al. [78] tested a nanoemulsion cream containing lavender essential oil and licorice extract for the healing of deep skin wounds in a rat model. The nanoemulsion revealed an increase in collagen deposition and a faster re-epithelialization as well as an increase in the antioxidant activity in the wound area. Hence, the preparation of nanoemulsions loaded with lavender essential oil and licorice extract is a promising strategy to be used in cosmetic products for cutaneous wound healing [78].

### 3.2. Liposomes

Liposomes can attach to the cell plasma membrane, mediating the release of its contents, demonstrating that they can be used for delivery purposes. For instance, p-chlorophenyl benzyl ether (CBE) is a potential candidate as a skin brightening agent, however, it cannot successfully pass the stratum corneum to reach the melanocytes located at the skin-deep layer due to its low solubility in water. Therefore, to overcome this limitation, Singpanna et al. [79] incorporated CBE into liposomes via the thin-film hydration method, leading to better skin penetration of the active ingredient. Liposomes were also able to improve the anti-melanogenic activity of CBE in B16-F10 cells [79]. Furthermore, the easiness in the synthesis of the liposomes as well as its efficiency in the encapsulation of active ingredients and constant release into the cells make them well suited for cosmetic preparation [64,80,81]. Indeed, liposomes are one of the most widely used cosmetic delivery systems since they can incorporate molecules with different characteristics, hydrophilic and lipophilic, into their aqueous core and nonpolar portion of the bilayer membrane, respectively [65]. In this way, the active compounds are protected from metabolic degradation [82]. This is particularly important for the transport of vulnerable agents such as vitamins [83], phenolic compounds [84], quercetin [85], and benzoyl peroxide [86,87]. Additionally, phosphatidylcholine, which is a major component of liposomes, is extensively used in skincare formulations since it contains softening properties. Recently, it was shown by Figueroa-Robles et al. [84] that the application of the liposomal technique allows for better penetration through the stratum corneum, preventing rapid degradation and acting as a control to regulate the release of phenolic compounds. Furthermore, it has been demonstrated to have the ability to deliver folate transdermally encapsulated into liposomes and incorporated in a cosmetic base without the need of a surfactant or external energy for permeation [88].

**Figure 3:**
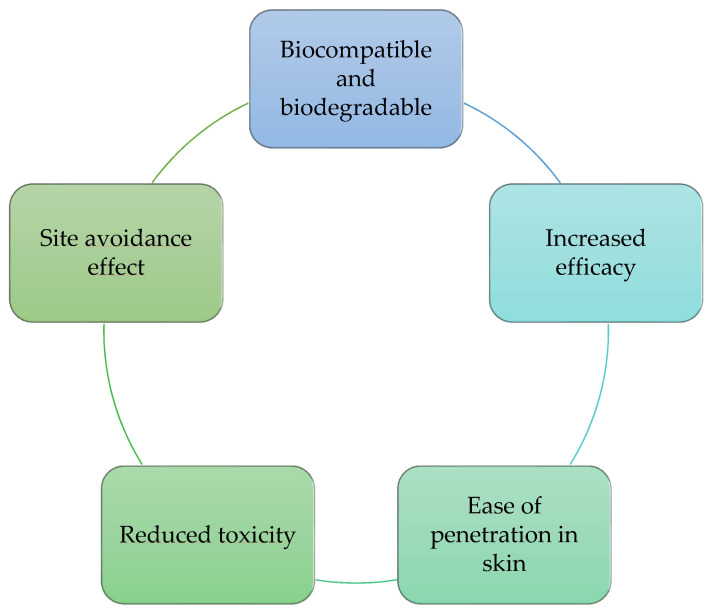
Positive aspects of liposomes.

Bi et al. [89] reported the use of liposomes to transdermally deliver vitamin D3. This new system resulted in a product with improved stability, which can be used to repair the photoaging condition. Despite the advantages of liposomes, as described in Figure 3, they also have some limitations in terms of their applicability. These limitations have hindered their widespread use in commercial products [90]. Among their limitations are the identifiable physical and chemical instability, low loading capacity, high production cost, low solubility, occasionally oxidation and hydrolysis reaction, osmotic sensitivity, and minimum reproducibility [64,90,91].

### 3.3. Solid Lipid Nanoparticles

**Figure 4:**
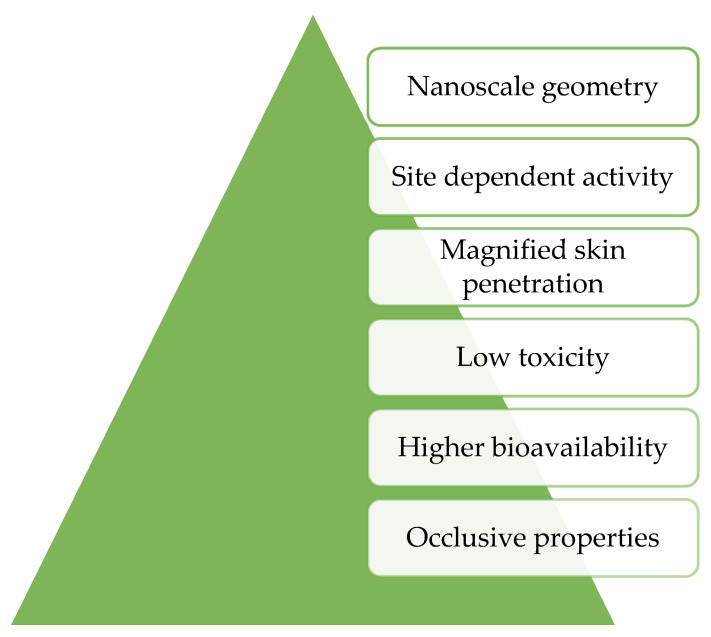
Beneficial characteristics of SLNs.

Solid lipid nanoparticles (SLNs) are biodegradable lipids with low toxicity [22,92] that can not only protect the constituents they incorporate from destruction, but can also be used as a means of transporting cosmetic agents into the stratum corneum since they can easily penetrate this layer [64,93]. SLNs can entrap various active compounds with several properties such as hydrophilic [94], lipophilic [95], or poorly water-soluble [96]. Furthermore, SLNs have better stability than liposomes as a result of being solid [97]. The encapsulation of these nanoparticles protects the cosmetic agents from biodegradation by enzymes, granting them transport in a controlled manner for a prolonged time and enhancing the penetration of the active agents into the stratum corneum [64,98]. Indeed, Soldati *i* verified that the incorporation of resveratrol into SLNs from natural seed butter *Theobroma grandiflorum* increased the antioxidant activity by 20% with improved permeation and retention of the active ingredient in the human skin [95]. Moreover, the SLNs containing resveratrol revealed an increase in resveratrol concentration more than 2-fold in the stratum corneum compared to a resveratrol ethanolic solution [95]. The intrinsic properties of SLNs such as nanoscale geometry, site-dependent activity, magnified skin penetration, low toxicity, and high bioavailability allow for their use in cosmeceuticals (Figure 4) [64]. Furthermore, these nanoparticles have occlusive properties that increase skin hydration [99]. The preparation method and physicochemical characteristics of the active agents can influence the efficiency of SLNs [100]. A study by Aland et al. [96] demonstrated that a transdermal delivery formulation of tazarotene loaded into SLNs had better tolerability than the marketed formulation for the treatment of psoriasis. Indeed, the new formulation did not reveal any sign of skin irritation, showing an improvement in transdermal delivery of tazarotene. In conclusion, several studies have demonstrated the effectiveness of SLNs in delivering active ingredients with different properties, which makes this technology of great value for the cosmetics industry.

### 3.4. Nanostructured Lipid Carriers 

**Figure 5:**
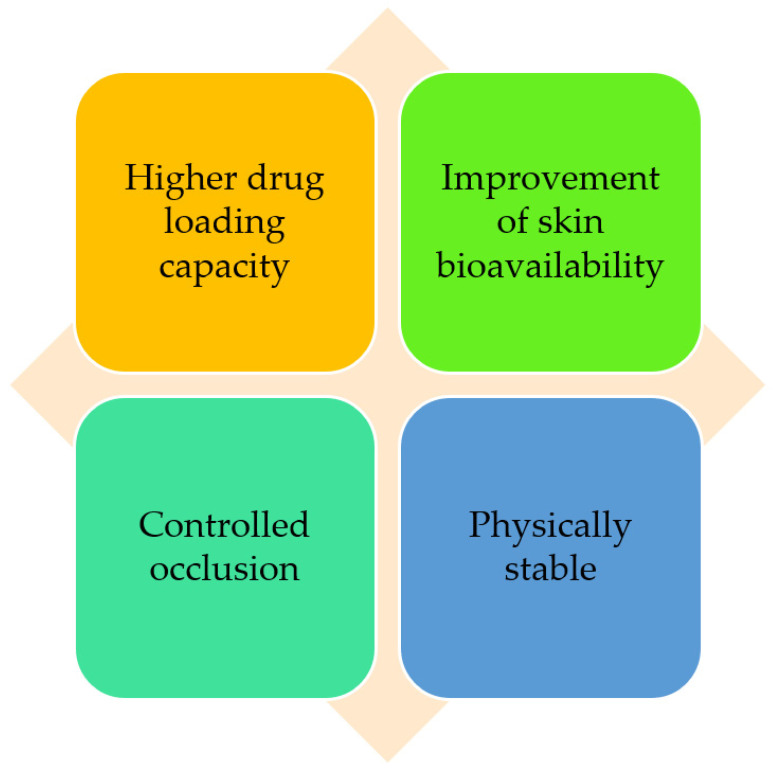
Attributes of NLCs.

Nanostructured lipid carriers (NLCs) were developed to solve the limitations related to SLNs, being better in terms of stability, skin hydrating ability, drug loading, penetration, sun protection feature, and safety [22]. These nanoparticles follow a biphasic release pattern and guarantee close contact to the stratum corneum, increasing permeability of the active substance through the skin. Furthermore, NLCs have enhanced UV protection with minimum side effects and are stable upon storage [22,101]. These NLC attributes including the improved skin bioavailability, film formation, and controlled occlusion make them an important asset to be used in cosmetics (Figure 5). Moreover, NLCs are formulated using physiological and biocompatible lipids, therefore, toxicity issues are reduced [65,102]. Noh et al. [103] developed a NLC-based transdermal formulation for isoliquiritigenin with ceramide with improved efficacy of the cosmetic agent. Quercetin, an antioxidant, can be used as sunscreen to provide additional skin photoprotection. Nevertheless, this antioxidant molecule has poor permeation and low stability, making it difficult to use in cosmetic products [104]. To overcome these limitations Felippim et al. [104] developed a photoprotective formulation containing quercetin loaded in NLCs. This formulation showed a significant improvement in the sun protector factor in vivo, without the need to increase the number of UV filters. In conclusion, the nanoencapsulation of quercetin revealed a synergistic effect in the sun protector factor with enhanced skin barrier function and hydration. Pereira et al. [105] proposed the encapsulation of clindamycin phosphate and rifampicin into NLCs to be used as a topical alternative for the treatment of hidradenitis suppurativa, which is a chronic inflammatory disease associated with a permanent obstruction of the pilosebaceous units. The formulation prepared revealed non-irritative behavior for topical application as well as drug-controlled release profiles. Furthermore, these nanocarriers provided a significant increase in rifampicin uptake into the hair follicles and the formation of a depot of clindamycin on sebaceous skin [105]. In brief, several works have revealed the ability of NLCs to overcome the limitations of different cosmetic ingredients and deliver these ingredients to the desired parts of the body, attaining long-term stability.

### 3.5. Niosomes

The building component of niosomes is a surfactant. Surfactants are non-toxic, biocompatible, and biodegradable, increasing the efficiency of niosomes [106]. Indeed, niosomes reveal high stability and are biodegradable. [107]. Furthermore, these nanocarriers enhance the bioavailability of poorly absorbed ingredients as well as improve skin penetration [108]. Indeed, niosomes allow the molecules to penetrate the living tissue at a high rate, which is a desirable feature in skin care applications and cosmetics [64]. A study by Lu et al. [109] reported the development of niosomes loaded with quercetin for whitening and antioxidant ability, which showed a better skin permeation, sustained release over time, and improved transdermal penetration with skin retention 2.95 times higher than that in the quercetin solution.

Radmard et al. [110] showed that a formulation of niosome containing arbutin is non-cytotoxic and does not cause skin irritation with improved skin delivery of arbutin. Therefore, arbutin loaded into niosomes could be a new approach for the treatment of hyperpigmentation conditions [110]. Several studies have demonstrated the advantage of using niosomes as nanocarriers in cosmetics [111,112,113,114].

### 3.6. Nanocapsules

Nanocapsules enable the release of active agents under controlled conditions and the prevention of harmful effects at the site of delivery due to their nanostructures [115,116]. Capsaicin is a topical analgesic used in the treatment of chronic pain, which is used for long periods and results in skin irritation [117]. Therefore, Contri et al. [118] showed that when capsaicin was encapsulated into nanocapsules, there was a decrease in the negative effects of capsaicinoids without changing their effect on skin. Moreover, the active ingredients were protected against photo- or chemical degradation, therefore increasing their stability. The enhanced efficacy of this system was due to the improved interaction with tissues and cells [116]. A recent study showed that UV filter nanocapsules with the organic UV filter octyl dimethyl para-aminobenzoic acid (OD-PABA) in mini-emulsion polymerization enhanced the sun protection factor by 300% compared with the free OD-PABA formulations. This study also reported that the encapsulation of OD-PABA could considerably minimize the cytotoxicity. It was observed that cell viability in the presence of UV filter nanocapsules improved 17%, increasing the safety of the UV filter in sunscreen formulations [119]. Other molecules have been successfully encapsulated into nanocapsules, forming promising formulations to be applied in the cosmetic field such as dutasteride (used in the treatment of alopecia) and desonide (used in atopic dermatitis) [120,121].

### 3.7. Nanospheres

Nanospheres can be loaded with several substances (drugs, enzymes, and genes) that are dissolved, encapsulated, or entrapped into the polymer matrix. The encapsulation protects them from chemical end enzymatic degradation [65]. Nanospheres are widely used in antiwrinkle, moisturizing, and anti-acne cream preparations to deliver the active ingredients to the deeper layers of the skin more efficiently and precisely [64]. A study by Müller et al. [122] reported the production of nanospheres formed by Ca^2+^ and polyP, aCa-polyP-NP, encapsulating retinol. These active ingredients activate collagen gene expression, which is desirable, for example, in antiwrinkle products since collagen decreases in aging skin. The Ca-polyP/retinol nanospheres increased cell growth and collagen type III expression. However, not many studies on the application of nanospheres as potential nanocarriers in the cosmetic field have recently been reported in the literature.

### 3.8. Gold and Silver Nanoparticles

The anti-microbial properties of nanogold and nanosilver particles are important for their use in the cosmetics industry. Indeed, it has been reported that silver nanoparticles used in cosmetics, for example, in underarm deodorant, will provide protection all day long. Likewise, nanogold could be added to toothpaste, since it eliminates the bacteria present in the mouth [98]. Concerning silver nanoparticles, their antibacterial activity is due to the modification in bacteria cell wall permeability as well as silver ions bonding to the respiratory chains. This association leads to an increase in the production of reactive oxygen species (ROS), making it difficult for the survival and growth of bacteria [64]. On the other hand, nanogold particles are biocompatible, non-cytotoxic, highly stable, and inert in nature. These nanoparticles can be easily delivered to the target site due to their shape, small size, crystallinity, and large surface area [123]. Moreover, the antioxidant and antibacterial properties of the gold nanoparticles enable the production of lotions that enhance skin texture and delay aging [115]. Nanogold particles have a very high drug-loading capacity and due to their powerful antibacterial and antifungal properties, they are considered a valuable material in the cosmeceutical industry [123]. In beauty care, gold nanoparticles are used to vitalize skin metabolism, grant antiseptic and anti-inflammatory properties, improve elasticity and firmness of the skin, and increase blood circulation [124]. Pulit-Prociak et al. [125] developed stable cosmetic formulations with silver or gold nanoparticles, proving their penetration in a skin model membrane. Additionally, both the silver and gold nanoparticle creams revealed suitable fungicidal properties [125]. Thus, the incorporation of these nanoparticles into creams or other products can be an interesting technique for the cosmetics industry.

### 3.9. Nanocrystals

**Figure 6:**
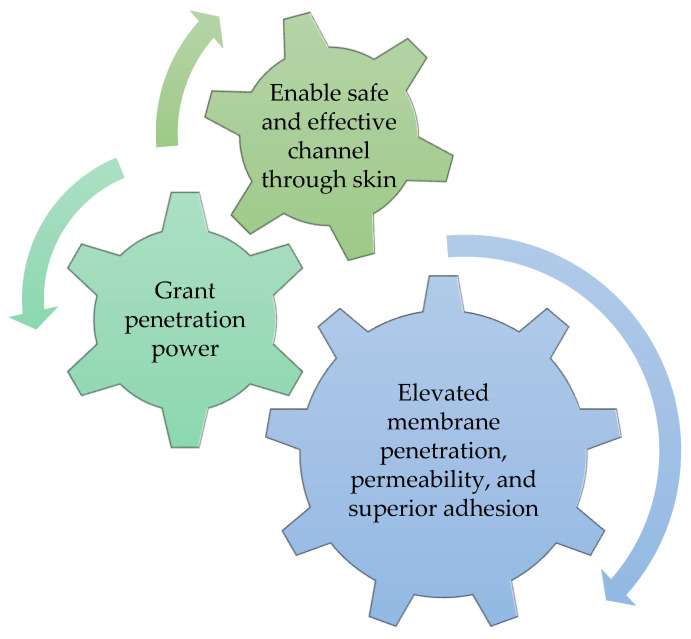
Properties of the nanocrystals.

Nanocrystals have interesting properties, particularly their significant adhesiveness, which leads to high retention time at the target site due to their high loading capacity and surface area [115]. Generally, nanocrystals are found as individual or polycrystalline forms incorporating rutin (flavonoids) as an active compound [126]. Furthermore, they have the required properties represented (Figure 6) [127] to make them suitable for the dermal application of cosmetics incorporating poorly soluble molecules [128]. Indeed, a study by Müller et al. [129] reported that both hesperidin and rutin nanocrystals boosted the sun protection factor, confirming that nanocrystals improve skin penetration into the skin. Quan et al. [130] prepared 18β-glycyrrhetinic acid (GA) nanocrystals to overcome its poor solubility in water and, consequently, its poor skin permeability and low bioavailability. GA is widely used in clinical for the treatment of skin inflammatory diseases. Its nanocrystallization has better water solubility and improved skin penetration. This formulation also provides the inhibition of pro-inflammatory factors and tissue edema in vivo [130]. Other examples of the application of nanocrystals have been reported in the literature as promising nanocarriers in cosmetics such as vitamin C and quercetin [131,132].

### 3.10. Dendrimers

**Figure 7:**
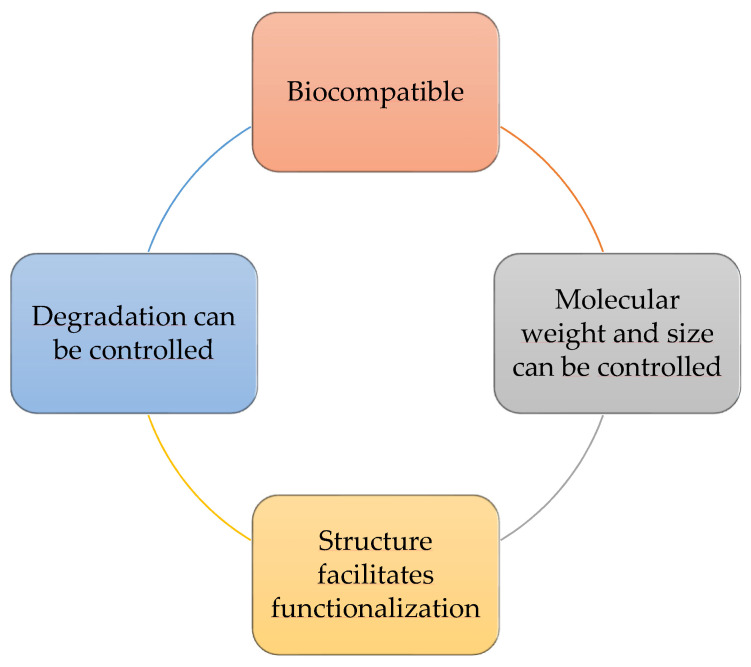
Advantages of dendrimers.

Dendrimers have end groups, which are engineered to attach active components aiming at a specific target. This characteristic results in the carrier’s versatility, as hydrophobic and hydrophilic drugs can be fused with the dendrimer [133]. Additionally, dendrimers have several positive characteristics for the cosmetics industry (Figure 7) [90] such as facilitating drug skin permeability and are used in skin and hair care products [115]. A recent study by Sanz del Olmo et al. [134] reported the synthesis of carbosilane dendrimers functionalized with phenolic acids (ferulic, caffeic, and gallic acids), revealing an improvement in the antioxidant activity and antibacterial capacity of polyphenolic compounds. Therefore, the application of dendritic systems as anchorage platforms for polyphenolic compounds in the cosmetics field would be advantageous [134]. Pentek et al. [135] developed a multifunctional dendrimer to increase the solubility and stability issues of resveratrol, leading to a scale-up and commercialization of the system as an anti-aging cream. In this way, the use of dendrimers as nanocarriers are a valuable strategy for the cosmetics industry.

### 3.11. Cubosomes

**Figure 8:**
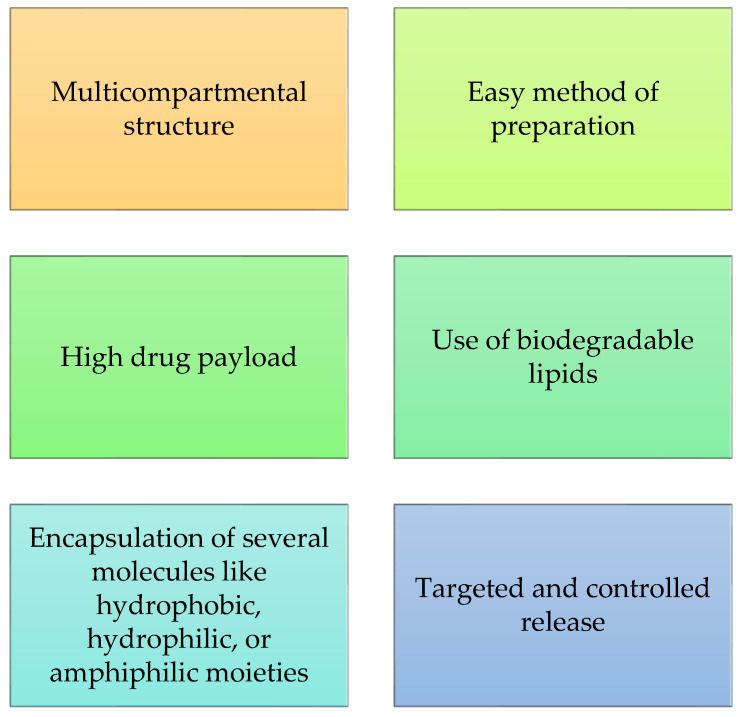
Properties of cubosomes.

Cubosomes are nanostructured particles that are thermodynamically stable, biocompatible, and have bioadhesive properties. These carriers can be used to deliver molecules by different routes such as topical, parenteral, transdermal, and oral. They have several properties such as a multicompartmental structure, easiness to prepare, high drug payload, use of biodegradable lipids, encapsulation of several molecules with different properties (hydrophobic, hydrophilic, or amphiphilic moieties), and the ability to target specific tissue and control release (Figure 8). Indeed, it has been shown that cubosomes can bring many benefits to the cosmetics industry. For example, Khan et al. [136] demonstrated that cubosomes loaded with erythromycin are effective for topical drug delivery in a sustained and non-invasive manner for the treatment and prevention of acne. Lately, not many studies on the application of cubosomes as potential nanocarriers in cosmetics have been reported in the literature.

### 3.12. Hydrogels

In the cosmetics industry, hydrogels can be used as skin delivery systems [64]. Pagano et al. [137] revealed a technological approach to stabilize lipoic acid, which is an anti-aging compound. Lipoic acid activity is limited by its low solubility, low stability to oxidation, and thermal processes. The stabilization was achieved by the intercalation of α-lipoic acid (ALA) in MgAl and ZnAl and subsequent introduction of the hybrid product in hydrogel formulations [137]. The study showed that the hydrogel containing MgAl-ALA had enhanced rheological properties and stability, was safer than ZnAl-ALA, and represents a valuable alternative to the commercial formulations available on the market [137]. Despite their suitable properties, not many recent publications on the application of hydrogels as potential nanocarriers in cosmetics can be found in the literature.

### 3.13. Fullerenes/Buckyballs

Fullerenes are an advanced type of nanoscale material that has an antioxidant potential superior to vitamins [64]. They control melanin production, having a brightening effect due to the inhibition of free radicals caused by UV exposure. Fullerenes are used in creams aiming to clear the dark circles surrounding the eyes, providing a healthy and fresh look [138,139]. A recent study by Saitoh et al. [140] showed that polyvinylpyrrolidone-entrapped fullerene (C_60_/PVP) significantly reduced the UVB-induced generation of intracellular peroxynitrite levels. Therefore, C_60_/PVP can be useful as a cosmetic ingredient to prevent skin injuries and dysfunction due to nitric oxide/peroxynitrite induced effects in human skin keratinocytes [140].

### 3.14. Polymersomes

Polymersomes are biologically stable and highly versatile carriers with the ability to encapsulate and release molecules that are easily altered through the addition of several stimuli-responsive and biodegradable blocks of copolymers. Their permeability, membrane thickness, and responsiveness to stimuli can be influenced by the synthetic block copolymers used to prepare them [141,142]. These nanocarriers can release the drug, in a controlled manner, as a result of their flexible membrane [143]. Oliveira et al. [144] revealed that polymersomes of Pluronic L121 have low photo/cytotoxicity, so they are a safe alternative to develop topical formulations, not only for application on UV exposed skin, but also for protein delivery. Moreover, it has been shown that antioxidant skin protection can be improved by catalase loaded Pluronic L121 polymersomes, especially in the deepest layers of the skin. Nevertheless, not many studies on the application of polymersomes as potential nanocarriers in cosmetics can be found in the literature.

### 3.15. Carbon Nanotubes

Carbon nanotubes (CNTs) have desirable qualities for application in the field of pharmacy such as lightweight, good tensile strength, and small size with a high aspect ratio [65]. This technology has also an important impact on cosmeceuticals, whereby various carbon nanoparticle patents have been filed such hair coloring and cosmetic products [90]. However, no recent studies on the application of CNTs in cosmetics have been found in the literature.

### 3.16. Nanosponges

Nanosponges are freely flowing particles with thin cavities (nanometer range) that can be filled with lipophilic and hydrophilic moieties. They have high entrapping potential and the ability to release the active ingredients in a controlled diffusion. Nanosponges can be loaded with antifungal, antibiotics, local anesthetics, etc. for topical use, having the relevant attributes to be used in dermatological and cosmetic products. These structures can be used to enhance lotions, gels, powders, creams, and ointments intended for topical use [65]. Kumar et al. [145] reported that the encapsulation of azelaic acid in nanosponges increased the efficacy of the drug concerning the solubility, release, and safety, with suitable antimicrobial, antityrosinase, and antioxidant activity for the treatment of hyperpigmentation associated skin disorders. Currently, not many studies on the application of nanosponges as potential nanocarriers in the cosmetic field have been reported in the literature.

## 4. Active Ingredients in Cosmetic Nanocarriers 

Active ingredients are the keys to the success of the cosmetics industry and their incorporation into nanocarriers, as mentioned in the previous section, is revolutionizing the industry. In order to understand the constraints associated with the active ingredients and their incorporation, in this section, we present their fragilities and how to overcome them.

Nanocarriers are usually used in the cosmetics industry to overcome the problems associated with bioactive agents such as low stability, poor solubility, and penetration ability. Most of the active ingredients are inherently unstable under environmental stress conditions. One of these ingredients is retinol as well as antioxidants that are sensitive to UV light, heat, and oxygen. As previously mentioned, encapsulation of these active ingredients can prevent their degradation, increasing their shelf-life and in vivo performance [65,146]. Indeed, a paper published in the literature reported that retinol, whose efficacy is limited by an extreme sensitivity to light and temperature, can be encapsulated by solvent evaporation using a cationic polymer with high efficiency [147]. This encapsulation allowed for the protection of the active ingredient from oxidation for at least eight weeks and showed that the release of retinol from the nanoparticles was not immediate, but released throughout 18 h [147]. Furthermore, nanocarriers allow for the use of poorly soluble molecules in different formulations by increasing their solubility [90,148]. For instance, lutein is unstable, has poor solubility, and bioaccessibility, however, its encapsulation in zein/tea saponin nanoparticles overcomes these limitations. In fact, these nanoparticles demonstrated great stability at pH 4.0–9.0, revealed excellent ionic strength stability and thermal stability as well as enhanced solubility and bioaccessibility of lutein [149]. Several active ingredients have been loaded into nanocarriers such as retinoids, antioxidants, enzymes, peptides, ceramides, hyaluronic acid, and organic UV filters [25], which lessen their use in cosmetics. The limitations of the main active ingredients are summarized in Table 2.

### 4.1. Retinoids

Vitamin A (retinol) and its derivatives (retinoids) are some of the most widely used and important active ingredients in the cosmetics industry. This is due to their ability to stimulate epidermal growth, differentiation, and maintenance as well as control sebum and enhance extracellular matrix production, reducing skin wrinkles and acne [25]. These ingredients are widely used in the treatment of pigmentation and photoaging disorders as they inhibit melanogenesis, increase cellular turnover, and block the transport of melanin to epidermal cells [156], although their usefulness can be limited (Table 2). In turn, the limitations can be overcome by using nanotechnological approaches. It has been reported that tretinoin (*all-trans* retinoic acid) and its precursor (retinal) performance can be enhanced by conjugating them with polymers that can self-assemble into nanoparticles [157,158]. The activity of retinoids can also be improved by their nanoencapsulation, as demonstrated by the decreased photodegradation of tretinoin into liposomes and caprolactone nanocapsules [159,160].

### 4.2. Antioxidants

Antioxidants have an important role in protecting DNA against damage caused by ROS produced by internal or external stimuli [161]. Antioxidant function is achieved through the neutralization of ROS, inhibiting ROS-producing enzymes or chelating transition metal ions. The excessive production of ROS in the skin and the consequent appearance of wrinkles and hyperpigmentation can be caused by the exposure to UV radiation. This aging can be prevented by the local administration of antioxidants, which may or may not be enzymes. Thereby, antioxidants can be incorporated into anti-aging products as well as in sunscreens, improving their photoprotective power [162]. Despite their benefits, antioxidants have limitations (Table 2) that can be overcome through the use of nanocarriers [151]. The nanocarrier is usually chosen depending on the polarity of the active ingredient [25]. A recent study by Maretti et al. [163] reported the nanoencapsulation of the highly lipophilic β-carotene in NLCs to promote skin permeation of this active ingredient across the stratum corneum barrier, enhancing its antioxidant properties in photoaging and epithelial skin cancer prevention. This investigation showed that NLCs approximately 200 nm in size could be an appropriate approach to overcome the stratum corneum barrier, aiming to provide deeper β-carotene actions as well as avoid its degradation [163]. Another work by Vieira et al. [164] reported the encapsulation of *Haematococcus pluvialis* carotenoids into poly-lactide-co-glycolide nanocapsules to overcome its low water solubility and elevated instability, resulting in an advanced topical product with antioxidant properties. Nanocapsules, which were prepared by the solvent displacement method, demonstrate the sustained release of carotenoids from a gel system resulting in a higher antioxidant activity than ascorbic acid. Therefore, the hydrogel developed can be of high value to the cosmetics industry since it may provide prolonged protection of the skin against the photooxidation process [164]. Other examples can be found in Table 3.

### 4.3. Enzymes

The utilization of enzymes in the cosmetics industry is relatively recent, being limited to a number of products available on the market. Enzymes have revealed a better performance compared to common active ingredients, however, their delivery in a cosmetic preparation is challenging (Table 2). Although their high molecular weight limits the skin penetration of enzymes, they have been recommended for skincare products including enzymatic antioxidants, DNA-repairing enzymes, hyaluronidases, lipases, and exfoliating enzymes [165]. Moreover, the use of enzymes in cosmetic products is expected to be enhanced by their nanoformulation [166]. For instance, the liposomal formulation of photolyase, referred to as photosomes, revealed the ability of the enzyme to repair UVB induced cyclobutane pyrimidine dimers in human keratinocytes [167]. Therefore, photosomes may be incorporated in sunscreens to reduce the development of skin cancer [167]. Other examples can be found in Table 3.

### 4.4. Proteins and Peptides

An important group of molecules used in cosmetics are short peptides comprising 2–7 amino acids that can be classified as signal peptides, neurotransmitter inhibitors, carrier peptides, peptides, and enzyme inhibitor peptides. These molecules are usually used in anti-aging products [168].

Currently, there are some non-toxic formulations for hair perming that were developed to substitute the current synthetic perming formulations. An example proposed by Cruz et al. [169] was based on the use of keratin decapeptide sequences derived from the human keratin genome. These peptides replace the harsh alkaline reductive solutions, minimizing or even avoiding the damage caused to the hair fibers by chemical products. In the work by Song et al. [170], the capacity of cysteine and polycarboxylic acids to substitute the thioglycolates and the hydrogen peroxide was demonstrated.

The use of peptides in topical formulations has drawbacks (Table 2) that can be overcome by their nanoformulation. For example, a study by Puig et al. [171] showed that the use of liposomes loaded with tripeptide-10 citrulline, chosen by its capacity to interact with collagen fibers, increased skin elasticity. A work by Suter et al. [172] reported the entrapment of a heptapeptide in SLNs, using shea butter as the dispersant phase and lecithin as a stabilizer. Hence, it was shown that SLNs allowed for peptide delivery into the skin, enabling it to perform protective functions [172]. Until now, the most efficient peptide delivery system is through nanoformulation over common formulations [25]. Other examples can be found in Table 3.

### 4.5. Ceramides

Ceramides are known constituents of the lipidic matrix, so their use as new moisturizing agents to restore the skin barrier functionality has been suggested. However, their application has some limitations (Table 2) that are usually overcome through the use of nanocarriers [152]. Indeed, microemulsions and nanoemulsions have been shown to encapsulate them very efficiently [173]. A study by Tessema et al. [174] reported the encapsulation of ceramides in lectin-based microemulsions and starch-based nanoparticles, revealing that microemulsions improved in vitro release and the penetration of ceramides compared to the other formulations. On the other hand, the nanoparticles retarded the release of ceramides and enabled the penetration of small quantities of ceramides into the deeper layers of the multilayer membranes. Both formulations were effective in concentrating ceramides in the stratum corneum [174].

### 4.6. Hyaluronic Acid

In young skin, hyaluronic acid is abundant, but it decreases with aging. Its ability to retain water molecules makes it widely used in cosmetics as a moisturizing agent [175]. It is also applied in anti-wrinkle products [176]. Nevertheless, the potential of hyaluronic acid can be limited (Table 2). Hyaluronic acid microinjection allows for maximum accumulation down to the dermal layer [177], but the use of nanotechnology has also been proposed to improve hyaluronic acid performance. For instance, Jegasothy et al. [178] demonstrated a superior penetration of hyaluronic acid by reducing its molecular weight and formulating the polymer in the form of nanoparticles.

### 4.7. Organic UV Filters

The chemical structure of organic UV filters is composed of a chromophore conjugated with an aromatic ring substituted by an electron-donating group [179,180]. The UV filters can be classified as UVA, UVB, or broad-spectrum absorbers. They are extremely important in the development of sun protection products. However, nowadays, they can be incorporated in different cosmetic products such as make-up to improve their value in the prevention of long-term UV induced damage. However, their use may cause concern (Table 2). The limitations of these active ingredients can be overcome by nanoencapsulation, which can not only optimize the properties of the carriers, but also increase the retention of the active ingredient in the uppermost layers of the skin, avoiding permeation [181]. Furthermore, the use of nanocarriers can solve common disadvantages of bioactive agents such as high lipophilicity and limited photostability. A recent paper by Daneluti et al. [182] reported the encapsulation of avobenzone, oxybenzone, and octyl methoxycinnamate in mesoporous silica SBA-15, revealing the increase in safety and efficacy of UV filters. Other examples can be found in Table 3.

**Table 3:** Applications of active ingredients into nanocarriers for cosmetics formulation.

Nanocarriers	Active Ingredients	Cosmetic Use	References
Polymeric micelles	Curcumin	Whitening	[183]
Nanostructured lipid carriers	*Passiflora edulis* seeds oil	Whitening	[184]
Niosomes	Quercetin	Whitening	[109]
Niosomes	Arbutin	Whitening	[110]
Nano sponges	Azelaic acid	Whitening	[145]
Nanostructured lipid carriers	Orobol	Anti-ageing	[185]
Nanoliposomes	Carnosine Palmitoyl tripeptide-5 Acetyl hexapeptide-3	Anti-ageing	[186]
Nanoemulsions	Astaxanthin	Anti-ageing	[187]
Dendrimers	Resveratrol	Anti-ageing	[135]
Solid lipid nanoparticles Nanostructured lipid carriers Nanoemulsion	Lutein	Anti-ageing	[188]
Nanostructured lipid carriers	Finasteride	Anti-alopecia	[189]
Nanoemulsions	Minoxidil	Anti-alopecia	[190]
Nanocapsules	Hinokitiol	Anti-alopecia	[191]
Lipid nanoparticles	Hinokitiol	Anti-alopecia	[192]
SLN-Silica particles	Octyl methoxycinnamate	Sunscreen	[193]
Nanostructured lipid carriers	Quercetin	Sunscreen	[104]
Gold nanoparticles	Snail slime	Sunscreen	[194]
Cellulose nanocrystals	Diethyl sinapate	Sunscreen	[195]
Nanoemulsions	Sunflower oil	Sunscreen	[77]
Nanocapsules	Octyl dimethyl para-aminobenzoic acid	Sunscreen	[119]
Liposomes Nanostructured lipid carriers Solid lipid nanoparticles	Avobenzone Omega-3	UV blocking sunscreen	[196]
Cubosomes	Erythromycin	Anti-acne	[136]
Microemulsions	Curcumin	Anti-acne	[197]
Microemulsions	Thai basil oils	Anti-acne	[198]
Liposomes	Lauric acid	Anti-acne	[199]
Keratin: Zein nanoparticles	Fragrances (linalool and menthol)	Hair cosmetic	[200]

## 5. Application and Efficacy of Active Ingredients in Cosmetic Nanocarriers

**Figure 9:**
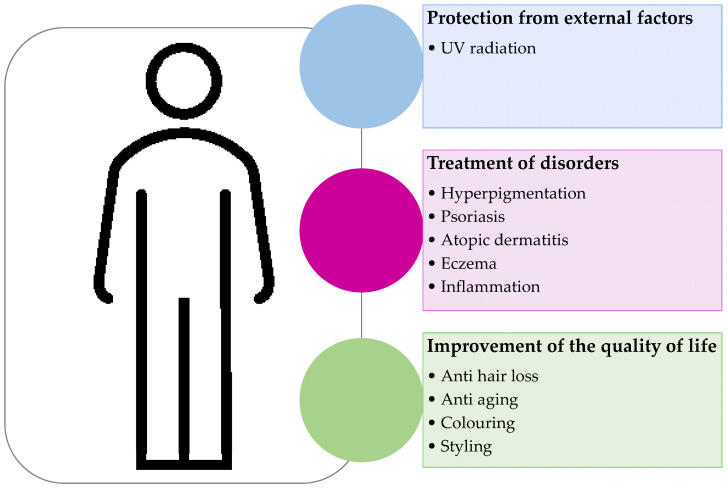
Advantages of the application of active ingredients in cosmetic nanocarriers.

The utilization of nanocarriers in cosmetic products enhances the solubility and stability of active components and overcomes the cuticle barrier effect. This effect allows the active cosmetics ingredients to enter the skin target site to perform its function in a controlled, sustained, and long-term release, hence solving several skin problems and skin diseases [62] and at the same time, improving the consumers’ quality of life (Figure 9).

The complications and diseases related to the epidermal barrier can lead to skin dehydration, which in turn leads to sensitive, dry, itchy, chapped skin. However, the skin barrier function can be restored, and the epidermal moisture content can be increased using nanocarriers incorporated with skin moisturizing components. Indeed, this technology can decrease skin problems, and at the same time, be preventive and have a therapeutic effect on chronic skin diseases such as atopic dermatitis, eczema, and psoriasis [201,202,203]. It has been reported that tacrolimus, which is a compound used to manage moderate to severe atopic dermatitis, can be loaded into mesoporous silica nanoparticles to overcome problems related to its solubility and effective topical delivery. The encapsulation of this compound revealed a significantly higher amount of retained tacrolimus and a much higher reduction in ear thickness, suggesting that this technique is a promising strategy for the topical delivery of hydrophobic drugs [204]. Another study published in the literature aimed to prepare and characterize an innovative nanoemulsion formulation loaded with *Linum usitatissimum* seed (linseed) oil (LSO) and investigate their potential in vitro and in silico evaluation for the treatment of atopic dermatitis. This paper demonstrated that LSO is a potential drug candidate for treatment of atopic dermatitis and its encapsulation in nanoemulsions allowed for an effective topical delivery [205]. A recent study has shown that NLC loaded with three active compounds (azelaic acid, white willow bark extract, and panthenol) has a prolonged moisturizing action and increases cellular viability, being an efficient strategy for the treatment of atopic dermatitis and acne [206]. Concerning psoriasis, a paper reported the incorporation of tacrolimus into lecithin chitosan hybrid nanoparticles by ethanolic injection technique. The prepared nanoparticles showed a higher skin deposition than the marketed product (63.51% vs. 34.07%) as well as a superior anti-psoriatic efficacy. In terms of in vivo drug deposition, the hybrid nanoparticles revealed superior skin deposition compared to the marketed product (74.9% vs. 13.4%) [207].

A whitening and freckle-removing effect can also be achieved using nanocarriers with whitening and freckle-removing efficacy components in cosmetics (Table 3). These components inhibit several pathways such as melanocyte proliferation, tyrosinase, and other related rapid-limiting enzyme activity as well as the inhibition of melanosome migration [208]. A ROS-responsive transdermal nanocarrier incorporating a whitening agent, glabridin, with the cell-penetrating peptide polyarginine R8 and bonded into the hollow mesoporous silica nanoparticles by the borate ester bond was developed. The work showed the rapid penetration of the synthesized nanocarriers through the epidermis reaching keratinocytes and melanocytes. There was glabridin release in a controlled ROS-responsive manner. A reversed UV-induced oxidative damage, phototoxicity, and decreased hyperpigmentation was also observed [209].

There are also anti-aging ingredients usually applied in cosmetics that can eliminate oxygen-free radicals. Nevertheless, there are some limitations such as low bioavailability, poor stability, decomposition under light, oxygen, and heat, and difficulty in transdermal absorption that can be overcome by the emergent development of nanocarriers with anti-aging components [210] (Table 3). A published paper reported the production of astaxanthin loaded nanoemulsions by a convenient low-energy emulsion phase inversion method. The prepared nanoparticles revealed an improvement in chemical stability and skin permeability to astaxanthin. Therefore, this technique might be a promising delivery system for the application of the active ingredient in dermal and transdermal products [187].

Hair loss can be treated by the topical application of anti-alopecia agents that can be loaded into nanocarriers, enabling the alleviation of the side effects of their direct application in the affected area [211] (Table 3). In the literature, a study reported on the development of nanoemulsions containing minoxidil to sustain and deliver active molecules to hair follicles for the optimization of alopecia areata treatment. Indeed, it has been shown that the synthesized nanoparticles penetrated hair follicles 26 times more efficiently than on the control sample, which means that they are a promising approach for the topical treatment of alopecia [190].

Sunscreen components can absorb and reflect UV light, hence reducing the occurrence of light linear disease and light aging. These active ingredients usually have some drawbacks such as large skin irritation and poor light stability, but these problems can be overcome by loading sunscreen efficacy components into nanocarriers (Table 3). A paper demonstrated the production of a new sunscreen formulation composed of hybrid SLN-silica particles loaded with octyl methoxycinnamate (Parsol^®^MCX), and their further incorporation into a hydrogel for skin administration. The enhancement of the bioadhesiveness of hydrogels, as a result of particles coated with colloidal silica, was evident. A synergistic effect of Parsol loading into SLNs in the increase in sun protection factor has also been shown [193]. Other examples are shown in Table 3.

## 6. Limitations of Nanocarriers 

Despite the benefits for skin health and clear efficiency of their use in cosmetic products, nanocarriers can have some disadvantages such as their higher development cost, sensitivity toward osmotic processes, low solubility, unsatisfactory stability, aggregation, low shelf life, and drug loading capacity as a result of hydrolysis and leaching [37]. The development of a formulation and subsequent preparation of a nanocarrier is not easy. There are a significant number of conditions that need to be taken into consideration such as the dosage and proportion of each component in the formulation. The preparation method is also very important as it can affect the internal structure, charge, stability, interaction with the active ingredients, and skin permeability [62]. Additionally, specialized equipment for the preparation of poor hydrophilic ingredients and uploading capacity due to partitioning effects may be necessary [37].

Some nanocarriers have toxicity potential. Their small size can be disadvantageous as it allows for the passage of these nanostructures through cell membranes, being able to reach organs and interfere with cells, proteins, and DNA [212]. For instance, nanocarriers smaller than 10 nm can act similar to gas disturbing the cell chemistry as they can cross cell membranes [213]. There are several routes through which nanocarriers can enter into human organisms: skin, respiratory, and gastrointestinal. Nanocarriers that enter the human body through inhalation can reach the brain [214]. These nanostructures have low encapsulation capacity, being necessary to increase the addition of surfactants in their formulations. In turn, surfactants can have adverse effects such as skin irritation and trauma, disruption of skin enzyme activity, causing abnormal body physiological function, and potential toxicity as a result of the accumulation of surfactants and nanocarriers in the body [215]. On the other hand, the permeation of nanocarriers into unhealthy skin may have distinct effects since the skin structure and its composition is different [216]. Therefore, further studies regarding the application of nanocarriers into unhealthy skin as well as long-term toxicity studies are needed.

The limitations of nanocarriers are not only related to scientific development but also to legislation. It is well-known that there are significant differences between the European and North American legislation that may hinder their application in the cosmetics field, even though there are defined regulatory and safety guidelines comprising toxicity and the labeling of nanocosmetics in the global market [217]. In the next section, we compare and discuss the differences between the cosmetics regulatory system in Europe and the USA.

## 7. Cosmetics Regulation in Europe and the USA 

Currently, there is no worldwide definition of a nanomaterial as a cosmetic ingredient. Therefore, every country follows its own description and legislation. Nevertheless, the Europe (EU) and North America (USA) are the two major markets for cosmetics products.

### 7.1. European Regulations

Nanocarriers or drug delivery systems are a new class of products—functional cosmetics.

In Europe, cosmetic products are regulated by the European Commission through the Cosmetics Regulation (EC No. 1223/2009). This directive offers the consumer a high safety level. When a cosmetic ingredient fulfils the criteria defining a nanomaterial as set out in the European Cosmetic Regulation, Article 2 (1) (k), it is necessary to notify the Commission. The information should comprise the nanomaterial identification, specification, toxicologic profile, and safety data (Art. 16 (3)) [218].

EC Regulation 1223/2009 describes the labeling rules for cosmetic products that have nanomaterials in their composition. It states that a nanomaterial must be undoubtedly identified in the ingredient list by using the word “nano” in brackets as a suffix to the compound name. According to European law, all cosmetics brands must have a responsible person (natural or legal) who is responsible for complying with this.

It is also mandatory to register each cosmetic, before reaching the market, at the EC Cosmetic Products Notification Portal (CPNP). It is important to mention that if the new formulation includes a new nanomaterial that did not go through a full risk assessment by the Safety Assessment of Nanomaterials in Cosmetics (SCCS), this fact must be transmitted to the EC. The information conveyed must include nanomaterial identification, physicochemical characterization, toxicity assessment, the safety of the cosmetic product, exposure conditions, and finally the estimated amount sold per year [219].

At the beginning of 2020, the European Union Observatory for Nanomaterials (EUON) declared that all companies involved with nanoforms should comply with REACH—Registration, Evaluation, Authorization, and Restriction of Chemicals regulations. The EC REACH regulations were issued in 2006 [220]. In 2018 (December), the EC restructured Regulation 1907/2006 to include nanoforms.

The necessity to reduce animal suffering and experimentation led the European bodies to instore new rules for the cosmetics industry. Therefore, no toxicological information regarding hazard identification can be obtained through animal use as it is strictly prohibited under EC Cosmetic Regulation No. 1223/2009. Therefore, different methods must be employed such as ex vivo and/or in vitro [56].

The EU Cosmetic Regulation suggests that nowadays, there is inadequate information on the risks associated with nanomaterials. To better assess their safety, the Scientific Committee on Consumer Safety (SCCS) should guide cooperation with relevant bodies on test methodologies that take into account the specific characteristics of nanomaterials (Articles 29 to 31 of the EU Cosmetic Regulation) [218]. This led the SCCS to publish the Guidance on the Safety Assessment of Nanomaterials in Cosmetics (SCCS/1484/12), which has been updated more than 20 times in the past 10 years. The last update occurred in March 2021 (SCCS /1618/20).

A report regarding the use on nanomaterials in cosmetics was published in July 2021 by the EC. According to data obtained from the Cosmetic Product Notification Portal (CPNP), on a daily basis, approximately 800 new cosmetic products enter the EU market upon their notification to the necessary organizations 10 of them with nanomaterials [26].

When analyzing the available data, regarding the notification of cosmetic products with nanomaterials per country, France (5%) has the highest number of notifications, followed by Poland (2%), Germany (1.5%), Italy (0.9%), and Spain (0.8%). When comparing the overall notifications in the EU of cosmetics with nanomaterials, France is still the major contributor, followed in this case by Italy, Germany, Spain, and Poland [221]. The Commission considers that these differences are due the divergences in the application of the law by national authorities and/or economic operators due to an ambiguous definition of nanomaterials and the reporting requirements.

According to Section 2 of the Commission Report (Review of Provisions Relating to Nanomaterials), the cosmetic definition of nanomaterials should be updated and presented in the next report. The main reason for this review is to highlight the differences found between the definition of nanomaterials in the Cosmetics Regulation and the 2011 Commission Recommendation.

### 7.2. USA Regulations 

In the USA, there is a lack of regulations concerning nanomaterials/nanocarriers in cosmetics. FDA is responsible for the monitoring of the use of nanoscale materials and nanotechnology in cosmetics, and also conducts and keeps abreast of related research.

The FDA does not have a legal definition for nanotechnology, although it is assumed that the term nanotechnology refers to materials with dimensions between 1 and 100 nm [222]. As a result, the FDA has formed the National Nanotechnology Initiative (NNI) and the Nanotechnology Task Force (NTF) to determine and evaluate the necessary regulations for nanotechnology products.

In the U.S., companies or individuals who want to market cosmetics are legally bound to ensure the safety of all the ingredients present in the product including the ingredients at a nanoscale level. Additionally, they are required to describe the conditions of use on the label. According to U.S. law, cosmetic products and ingredients do not require FDA approval, not even pre-market approval [222]. In contrast to European law, the FDA does not require a clear indication on the label if any of the ingredients are nanomaterials. They argue that the particle size may not necessarily be involved in the toxicologic profile, which can confuse consumers.

Even though there is a significant lack of regulation by the FDA, there are some regulations and protocols that cosmetics producers can select to follow. The FDA and the Personal Care Products Council (PCPC) have established protocols for the registration of ingredients and description of any adverse reactions, even though this is not mandatory, but voluntarily, as the name clearly states—Voluntary Cosmetic Registration Program (VCRP). Using this platform, cosmetic manufacturers can analyze the materials that pose risk and remove them from the final product [219].

**Figure 10:**
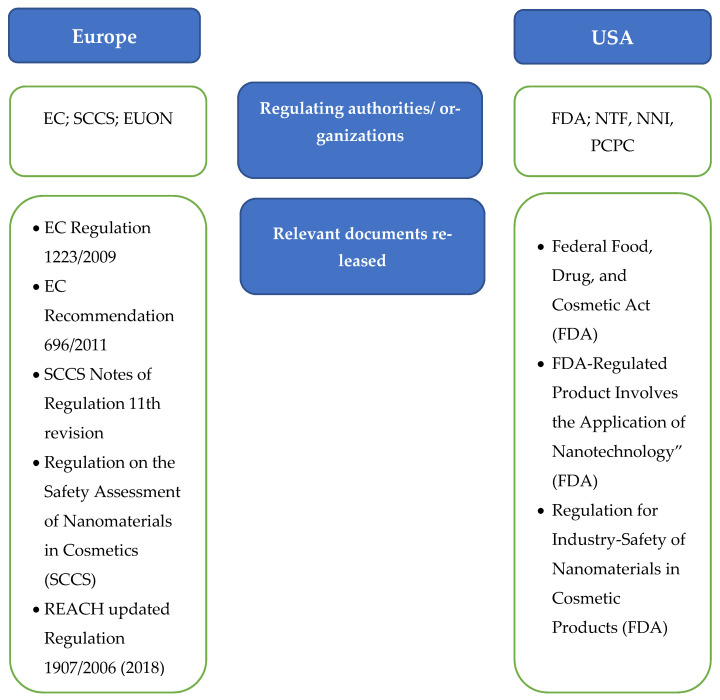
Regulatory bases for cosmetic products comprising nanomaterials in the EU and USA. Abbreviations: EUON: European Union Observatory for Nanomaterials; EC: European Commission; NNI: National Nanotechnology Initiative; NTF: Nanotechnology Task Force; REACH: Registration, Evaluation, Authorization and Restriction of Chemicals; SCCS: Scientific Committee on Consumer Safety; FDA: Food and Drug Administration; PCPC: Personal Care Products Council.

Taking into consideration that the regulatory authority for cosmetic products does not allow for cosmetics or their ingredients to be changed or mislabeled, the FDA has published a safety guide entitled “Guidance for Industry: Safety of Nanomaterials in Cosmetic Products”. This guide refers to issues related to nano-tech strategies and the usage of nanomaterials in cosmetics products [222]. Finally, the Guidance for Industry has information regarding the safety assessment of nanostructured constituents in cosmetic formulation and was developed to help identify any possible safety issues and how to evaluate them [23] (Figure 10).

### 7.3. Other Countries 

Before briefly discussing other countries, it is important to present another concept, which is that of cosmeceuticals. Cosmeceutical consumption products are at the frontier of cosmetics and pharmaceutical products. Although, this term is not recognized by the Federal Food, Drug and Cosmetics Act and FDA, it is known that several cosmeceuticals modify skin physiological processes, but producers evade clinical trials by presenting specific claims to escape the expensive and long authorization process by FDA. The cosmetics industry is facing new challenges every day. These challenges require stricter regulation in order to guarantee the safety of the marked products. A new group is being created by different countries to adjust cosmeceuticals or borderline products (Table 4).

## 8. Conclusions and Future Perspective

The skin aspect is of utmost importance both physiologically and psychologically. This fact has driven the boom of the cosmetics industry for the past years. It is well-known that the revenue involved in this market is extremely high. It is expected to reach over USD 800 billion by 2023. By itself, the nanotechnology market is one of the most promising fields, but when combined with the high-value cosmetics market, it has led to a technological revolution with a growth rate per year of 17%. The possibility of incorporating the active ingredients of cosmetics in new and improved nanocarriers such as liposomes, niosomes, and cubosomes among others, has resulted in a significant increase in the effectiveness of the products. There are several advantages in the incorporation of cosmetic bioactive ingredients into new and improved carriers, namely, high stability, biocompatibility, controlled drug release, and high drug loading capacity, among others.

No doubt that the use of these carriers in the cosmetics industry is highly important, but there are also significant challenges ahead, particularly in terms of biosafety and polymer immunogenicity.

The novelty in this field and the good results should be analyzed carefully. The number of reports regarding the effect of these nanoparticles on metabolic pathways and metabolites kinetics is scarce. Therefore, it is necessary to conduct in-depth research on the long-term effects.

The lack of coordinated regulatory guidelines throughout several countries also poses a risk to the safety evaluation of cosmetic products. The differences between the European regulations and the USA regulations are significant. While in the USA, the registration of the ingredients, particularly nanomaterials, is not mandatory, in Europe, it is absolutely necessary to register all of the ingredients, with special attention to the nano ones.

More recently, the U.S. FDA has recommended human safety studies to evaluate the post-marketing safety data.

The main concerns regarding the safety of nanomaterials in cosmetics were raised in Europe, which led to the development of stricter regulations.

Shortly, it is expected that the cosmetics field will carry on to develop new and more effective products based on nanomaterials. It is anticipated that the legislation in the USA will approach the harsher European regulations.

“Flawless skin is the most universally desired human feature”–Desmond Morris.

## Figures and Tables

**Table 1:** Compositions and sizes of carriers used in the cosmetics industry.

Carriers	Composition	Size Range	References
Nanoemulsions 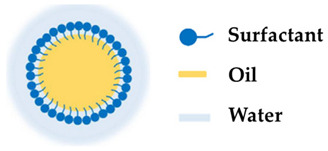	Nanoscale droplets comped by oil, surfactant, water cosurfactant	50–200 nm	[22,64]
Liposomes 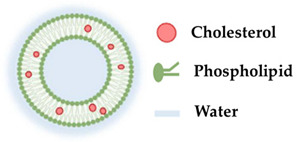	Vesicular structures with an aqueous core enclosed by one or more bilayer membranes composed of natural or synthetic phospholipids. Liposomes also have cholesterol in their composition	20 nm–2 µm	[64,65]
Solid lipid nanoparticles 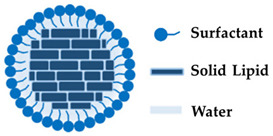	Composed by a shell of a single layer with a lipoic core made from complex glyceride mixtures, purified triglycerides, and waxes; They are stabilized by polymers or surfactants	50–1000 nm	[22,65]
Nanostructured lipid carriers 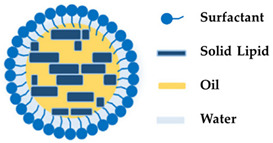	Structures that present an aqueous and oily phase	10–1000 nm	[66]
Niosomes 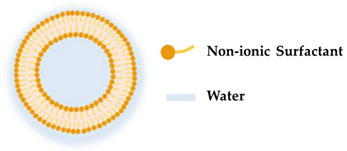	Vesicles with a bilayer structure synthesized from nonionic surfactants possessing hydrophilic and hydrophobic terminals; are conjugated with cholesterol and polyethene glycol	100 nm–2 µm	[22,65]
Nanocapsules 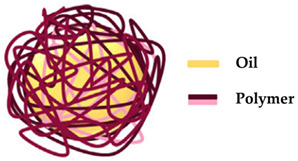	They have a solid and liquid core, where the active ingredients are protected and entrapped by a polymeric membrane which can be natural or synthetic	100–500 nm	[65,67]
Nanospheres 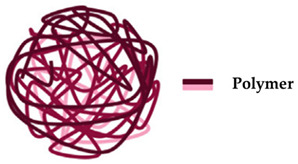	Crystalline or amorphous spherical nanoparticles having a core-shelled structure	10–200 nm	[64]
Nanogold 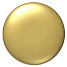	Nanoparticles of gold	5–400 nm	[64]
Nanosilver 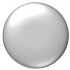	Nanoparticles of silver	1–100 nm	[64]
Nanocrystals 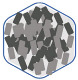	Aggregates of atoms that combine into a “cluster” (Chavda)	10–400 nm	[65,68]
Dendrimers 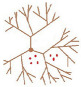	Three-dimensional synthetic polymers are micellar, globular, radially symmetric and monodisperse.	2–200 nm	[64]
Cubosomes 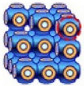	They are prepared by self-assembly of liquid crystalline particles of some surfactants when combined with water and a microstructure at a specific ratio; Microstructure of cubosomes is obtained by the ratio of water, surfactant system and aqueous lipids	10–500 nm	[64,69]
Hydrogels 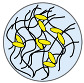	Polymeric networks with physical and chemical cross-links, expanding without dissolving in water or other biological fluids.	10–100 nm	[65,70]
Fullerenes/Buckyballs 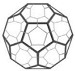	They are composed by 60 atoms of carbon	0.4–1.6 nm	[71,72]
Polymersomes 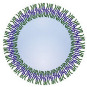	They are formed by self-assemble copolymer amphiphiles blocks, consisting in artificial vesicles which have an aqueous cavity in the center	100 nm–few µm	[65,73]
Carbon nanotubes 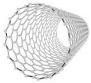	Empty cylindrical fibers formed by graphene walls that are rolled “chiral” angles	0.7–50 nm	[65]
Nanosponges 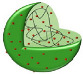	These are free-flowing particles and have a 3D network of degradable polyester	<1 µm	[64,74]

**Table 2:** Limitations of the active ingredients.

Active Ingredient	Limitations	References
Retinoids	Chemical structure exposes the retinoids to photopolymerization, photodegradation, photooxidation and photoisomerization; Some of them cause sensitization and skin irritation	[150]
Antioxidants	Limited stability in topical preparations	[151]
Enzymes	The native structure is destabilized by many common ingredients, which strongly affects enzymatic activity; their high molecular weight limit enzymes skin penetration	[25]
Peptides	Susceptible to degradation and low permeability	[25]
Ceramides	Low solubility	[152]
Hyaluronic acid	Hyaluronic acid with high molecular weight and hydrophilicity reveals poor penetration	[153]
Organic UV filters	May cause many adverse effects due to the production of toxic metabolites and ROS, which can be triggered by percutaneous accumulation and absorption	[154,155]

**Table 4:** Definition of cosmeceuticals and rules in some countries.

Country	Definition	Rules	References
**Japan**	Product that are not a cosmetic or a drug, is a “quasi-drugs”	Ingredients need to be pre-approved before including them into the “quasi-drugs” classification and require pre-approval before introduced them into the market	[223]
**Korea**	Korea Food and Drug Administration (KFDA) classifies them as “functional cosmetics”	KFDA is responsible for improving the safety and evaluation of functional cosmetics	[224]
**Thailand**	According to the used ingredients in cosmeceuticals, they are classified as “controlled cosmetics”	the notification from the FDA for the use of this products is mandatory	[225]
**New Zealand**	The category in which cosmeceuticals are accommodated is called “related products”	The regulation applied in New Zealand is similar to the EU legislation. The specifics of claims regulation and nanomaterials are the same and must be notified to Environmental Protection Authority (EPA)	[226]
**Australia**	In Australia, goods can be categorized based on claims about the product and product composition; the borderline products are classified as “therapeutic goods”	Only approved ingredients are used for the manufacture of these products. The Australian Register of Therapeutic Goods is the organization that registers "therapeutic goods".	[227]
**USA**	In the U.S., there are three categories: cosmetics, drugs, and over-the-counter medications. There is not a legal definition of cosmeceuticals according to FDA.	Classification by the U.S. FDA depends on the claims of the products.	[222]
**European Union**	The EU does not have a category to be called cosmeceuticals, but it has stringent law in which any claims made by the company are required to be submitted as a proof	The European regulation requires that cosmetic manufacturers declare all the nanoparticles/nanomaterials present in their products. They are required to add the word nano to the ingredient list. Regulation (EC) No.1223/2009.	[26]
**China**	“cosmetics for special use”	Similar to the FDA, but more rigorous, the China Food and Drug Administration (CFDA) requires that all foreign cosmetic products, before their release into the Chinese market, perform a safety evaluation comprising of several tests such as microbiology, toxicity, long-term toxicity, and carcinogenic. The manufacturers are also required to conduct trials to ensure their safety for humans. The cosmetics (imported) are divided into two main categories: special use cosmetics and ordinary ones. As a result, each category needs a distinct type of permit from the State Food and Drug Administration (SFDA). Finally, the Health Administration Department of the State Council—SFDA—must issue hygiene or record-keeping permit for the marketing of cosmetics.	[228]

## Data Availability

Not applicable.
